# Successful Treatment of Polyarteritis Nodosa With Multifocal Intracranial and Mesenteric Stenoses Using Endovascular Stenting and Tocilizumab

**DOI:** 10.7759/cureus.89607

**Published:** 2025-08-08

**Authors:** Mariana Nobre, Miguel Carrilho, Angelo Dias, Mariana Dias, Joana Martins

**Affiliations:** 1 Department of Internal Medicine, Local Health Unit of Santa Maria, Lisbon, PRT; 2 Department of Neuroradiology, Local Health Unit of Santa Maria, Lisbon, PRT; 3 Department of Neurology, Local Health Unit of Santa Maria, Lisbon, PRT; 4 University Clinic of Internal Medicine I, Faculty of Medicine, University of Lisbon, Lisbon, PRT

**Keywords:** endovascular procedures, il-6 receptor antagonist, intracranial artery stenosis, mesenteric vasculitis, polyarteritis nodosa

## Abstract

Polyarteritis nodosa (PAN) rarely affects both intracranial and mesenteric arteries. Evidence on optimal timing of revascularisation and the role of interleukin-6 blockade remains limited. A 73-year-old man with longstanding ankylosing spondylitis presented with weight loss and elevated inflammatory markers. Imaging revealed multifocal stenoses in the basilar, anterior, middle and posterior cerebral arteries, alongside a critical 4-cm superior mesenteric artery (SMA) stenosis. Temporal artery histology was negative; anti-neutrophil cytoplasmic antibodies (ANCA) and viral serologies were also negative. A diagnosis of systemic PAN was made. High-dose glucocorticoids and cyclophosphamide normalised inflammatory markers, but severe post-prandial abdominal pain persisted. Endovascular SMA stenting, performed during biochemical remission, resolved the angina and enabled weight recovery. Two years later, the patient developed a lacunar stroke alongside an inflammatory flare and worsening intracranial stenoses. Monthly tocilizumab induced rapid clinical and serological remission, sustained for 18 months. This case highlights that concomitant intracranial and mesenteric stenoses signal high-risk PAN. Additionally, deferring revascularisation until remission improves outcomes, and tocilizumab is a viable steroid-sparing option after cyclophosphamide failure, including cases of PAN with central nervous system involvement.

## Introduction

Polyarteritis nodosa (PAN) is a systemic vasculitis that primarily involves medium-sized arteries and is characterised by necrotising inflammatory lesions leading to focal aneurysms, stenoses and segmental arterial occlusions [1]. PAN most commonly affects men between 40 and 60 years of age and is typically idiopathic [1,2]. Unlike other systemic vasculitides such as microscopic polyangiitis or granulomatosis with polyangiitis, PAN involvement is confined to the arterial circulation, sparing veins, venules and capillaries; it is typically negative for anti-neutrophil cytoplasmic antibodies (ANCA) and does not cause glomerulonephritis or pulmonary infiltrates [2].

PAN usually presents with constitutional symptoms and frequently affects the medium-sized arteries of kidneys, skin, gastrointestinal tract and peripheral nervous system, most often as mononeuritis multiplex due to nerve infarction [2]. Central nervous system (CNS) involvement is rare, reported in only 4.6% of cases today, although historical series described higher rates in advanced disease reaching 20%-40% [3]. Central neurological manifestations are usually caused by ischaemia due to inflammation and occlusion of deep perforating branches (such as the lenticulostriate arteries) often resulting in lacunar syndromes and only rarely by stenoses of the large intracranial vessels in the circle of Willis [4]. Gastrointestinal involvement, present in up to 50% of patients, may manifest as post-prandial abdominal pain or frank intestinal ischaemia from mesenteric stenosis, the latter being a major cause of morbidity and mortality [2,5].

## Case presentation

A 73-year-old man with ankylosing spondylitis (AS), a permanent pacemaker for sick sinus syndrome, and a known occlusion of the left vertebral artery was referred to the Internal Medicine outpatient clinic for unintentional 10 kg weight loss over three months (14% body weight), anorexia and asthenia, without features of active AS. Laboratory tests showed normocytic normochromic anaemia (9.8 g/dL), leucocytosis (16 400×10⁹/L), thrombocytosis (530 000×10⁹/L), markedly elevated erythrocyte sedimentation rate (ESR > 120 mm/h), hyperfibrinogenaemia (756 mg/dL), raised C-reactive protein (CRP 15 mg/dL) and hyperferritinaemia (1 171 ng/mL).

**Figure 1:**
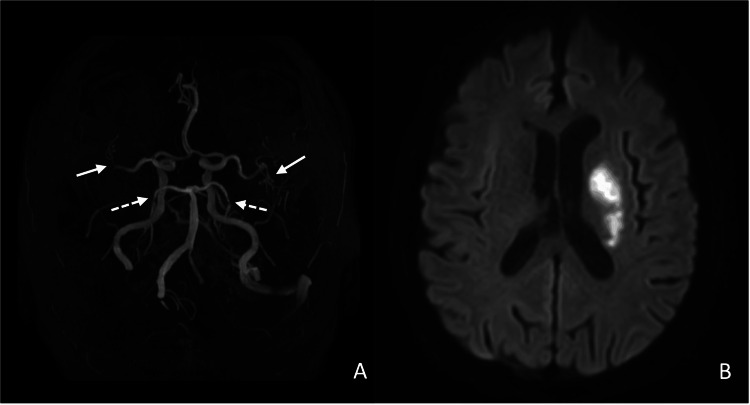
Intracranial stenoses and acute ischaemic stroke in polyarteritis nodosa (A) Magnetic resonance angiography (MRA) of the intracranial vasculature demonstrates multiple marked stenoses in the proximal segments of both the middle (full arrows) and posterior (dashed arrows) cerebral arteries. (B) Axial diffusion-weighted imaging (DWI) demonstrates an acute ischaemic lesion involving the left lentiform and caudate nuclei, as well as the corona radiata, within the vascular territory of the perforating branches of the left middle cerebral artery.

The patient underwent a CT scan of the chest, abdomen and pelvis; a PSA test; and a colonoscopy as part of the investigation to exclude occult malignancy. During follow-up, he developed bilateral frontotemporal pressure-type headache and bilateral inflammatory knee pain. Transcranial Doppler revealed the known occlusion of the left vertebral artery and new multifocal intracranial stenoses: moderate basilar stenosis, severe posterior cerebral artery stenosis and mild bilateral stenoses of the middle and anterior cerebral arteries, also evident in the magnetic resonance angiography (Figures 1A, 1B). Suspecting vasculitis, the patient was started on prednisolone 1 mg/kg, which normalised ESR, CRP and other acute-phase markers and corrected the anaemia. Temporal artery Doppler showed no halo sign, and temporal artery biopsy (performed two weeks after corticosteroid initiation) was negative for giant cell arteritis. Brain MRI demonstrated diffuse microvascular leukoencephalopathy and multiple sub-centimetre lacunar sequelae in the right parietal subcortical region, right periatrial area, forceps minor, adjacent to the frontal horn and the left lateral ventricle. ANCA, cryoglobulins and hepatitis B/C serology were negative; electromyography excluded peripheral neuropathy.

**Figure 2:**
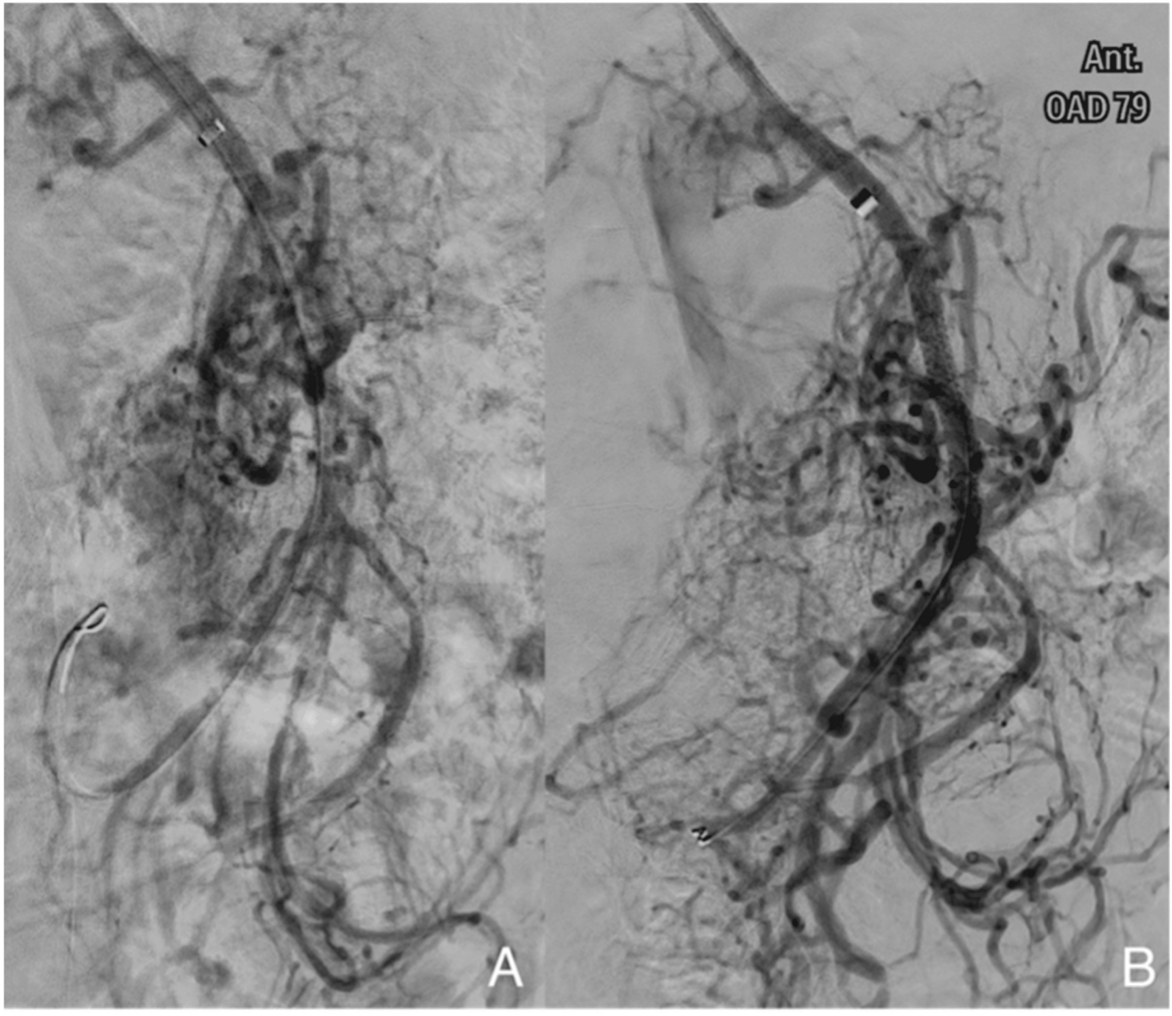
Digital subtraction angiography findings: pre- and post-stenting of superior mesenteric artery stenosis (A) Baseline image showing extensive collateral circulation suggestive of high-grade stenosis of the superior mesenteric artery (SMA). (B) Post-stenting image demonstrating improved anterograde flow through the SMA.

Three weeks later, the patient developed post-prandial periumbilical pain, alimentary vomiting and further weight loss. CT angiography showed a severe 4-cm stenosis of the superior mesenteric artery (SMA) beginning 2.5 cm from its origin, with circumferential wall thickening suggestive of medium-vessel arteritis. With probable PAN involving a target organ, worsening intestinal angina and progressive weight loss, he was admitted and received three pulses of methylprednisolone 1 g/day and the first IV cyclophosphamide pulse 600 mg according to CYCLOPS protocol [6]. Although inflammatory markers normalised, disabling angina persisted. After discussion with vascular surgery, SMA angiography with stent placement was performed, achieving an excellent result (Figures 2A, 2B) with complete resolution of intestinal angina and weight recovery. Dual antiplatelet therapy was continued for one year, followed by single, as per standard post-stenting protocol. Ten outpatient cyclophosphamide pulses were completed, and maintenance therapy was azathioprine 125 mg and prednisolone on a tapering course, although unable to reduce the dose below 12.5 mg/day.

Two years later, the patient was readmitted with an ischaemic stroke in the left middle cerebral artery territory (Figure 1B), presenting with aphasia, central facial paresis and right hemiparesis for an initial National Institutes of Health Stroke Scale (NIHSS) [7] score of 7; reperfusion therapy was not indicated. A full etiological workup for the stroke was performed, including a 24-hour Holter monitoring which did not reveal atrial fibrillation or other significant arrhythmias. Inflammatory markers rose again, with serum interleukin-6 increasing to 43 pg/mL. Concurrently, intracranial stenoses progressed. A five-day course of IV methylprednisolone 1 g/day was given, and monthly IV tocilizumab was started, achieving rapid clinical and biochemical remission that has been sustained for 18 months. Clinical remission was defined as resolution of constitutional symptoms, post-prandial intestinal angina and absence of new neurological deficits, accompanied by stable weight regain. Biochemical remission required sustained normalisation of acute-phase reactants (ESR < 20 mm/h, CRP < 5 mg/L, fibrinogen < 400 mg/dL) plus correction of anaemia and thrombocytosis. Transfer to a rehabilitation unit enabled modest improvement in his focal deficits, resulting in a follow-up NIHSS score of 5.

## Discussion

PAN is diagnosed by a combination of clinical, laboratory, imaging and histological criteria, requiring evidence of systemic inflammation with medium-sized arterial involvement and exclusion of other vasculitides such as ANCA-associated forms [8,9]. Conventional angiography remains the gold standard, although CT or MR angiography is a viable non-invasive alternative [8]. In this patient, imaging confirmation of multifocal intracranial stenoses and critical SMA stenosis supported the diagnosis and represents a rare pattern of multi-organ PAN involvement. We undertook a focused review of the literature on multi-territorial intracranial stenosis and mesenteric involvement in PAN and on contemporary immunosuppressive and endovascular approaches.

CNS disease in PAN is rare and heterogeneous, most often presenting as diffuse small-vessel leukoencephalopathy, cerebral ischaemia secondary to arterial occlusion, or, rarely, subarachnoid or intracerebral haemorrhage from aneurysm rupture. Multifocal lacunar strokes typically occur in territories supplied by medium-sized perforating branches, especially the lenticulostriate arteries, which perfuse the basal ganglia and internal capsule [3,4]. Simultaneous multifocal stenoses of the anterior, middle and posterior cerebral arteries, as in this case, are exceptional. Additional stroke mechanisms include severe hypertension and high-dose corticosteroid therapy [3,4].

To date, we have located only three further reports describing multifocal intracranial stenosis in PAN, all drawn from the GLOBAL-PAN cohort. Two showed occlusion of circle of Willis branches together with subclinical mesenteric disease, whereas the third exhibited isolated lacunar cerebral ischaemia. These observations reinforce that this vascular phenotype, although rare, seems to correlate with higher inflammatory activity and an earlier need for intensive immunosuppression [10].

Regarding differential diagnosis, giant cell arteritis (GCA) must be considered; it typically affects extracranial branches of carotid arteries, including the superficial temporal and ophthalmic, alongside subclavian arteries and aorta [11], with mesenteric stenosis being rarely reported [12]. Also, strokes are rare but preferentially occur in the vertebrobasilar circulation [11]. The negative temporal artery biopsy (despite steroid therapy) and absence of jaw claudication, visual symptoms or polymyalgia rheumatica made GCA unlikely.

Overall, PAN best matched the totality of findings. Concurrent involvement of anterior and posterior cerebral territories, including a lacunar stroke, associated with concomitant SMA stenosis is more typical of PAN [2,4]. Vascular manifestations in AS are uncommon; vasculitis is more often described as chronic aortitis of the ascending aorta [13]. PAN associated with AS is extremely rare and poorly understood, with only a few cases reported. A causal relationship is unproven and may represent coincidental comorbidity or secondary immune activation [14].

Regarding therapy, SMA revascularisation was justified by refractory symptoms and malnutrition risk. Although open surgery offers greater durability, endovascular revascularisation is preferred in high-surgical-risk patients because peri-procedural morbidity and mortality are lower [15]. Long, tubular vasculitic lesions may yield poorer technical results, and stents can exacerbate vascular inflammation, increasing restenosis or stent failure, although long-term outcome data remain limited [9]. Ideally, intervention should be performed in clinical and biochemical remission with ESR/CRP control [16].

Cyclophosphamide remains the first-line agent to induce remission in PAN with severe organ involvement, but interleukin-6 blockade with tocilizumab is gaining traction for refractory or relapsing disease [17]. Tocilizumab was preferred because the patient’s serum IL-6 was markedly elevated and the drug offers remission rates comparable to anti-tumour necrosis factor (TNF) or rituximab. A 2024 retrospective European collaborative study reported complete remission in 50% of patients treated with tocilizumab, compared with 40% for anti-TNF agents and 33% for rituximab [18]. While tocilizumab, as a large monoclonal antibody, is not expected to readily cross the blood-brain barrier, it has been successfully used in various vasculitides with CNS involvement since it controls systemic inflammation [19,20]. Although the evidence base is still limited, these data support IL-6 blockade as a viable steroid-sparing strategy when cyclophosphamide or anti-TNF agents fail [18]. Historically, PAN carried high mortality (five-year survival 40%-60%), but prognosis has improved markedly with early recognition and immunosuppressive therapy, reaching 80% five-year survival in specialised centres [9]. Nonetheless, severe GI and CNS involvement, as in this patient, remain poor prognostic markers [2].

## Conclusions

Concomitant multifocal stenosis within the circle of Willis and critical SMA involvement is an exceptional PAN phenotype and portends higher morbidity. This case reinforces the importance of early vascular imaging in PAN patients with atypical CNS or gastrointestinal symptoms. Timely endovascular revascularisation, performed after inflammatory control, can be lifesaving in refractory intestinal angina. IL-6 inhibition may be considered as a steroid-sparing strategy in PAN cases with relapsing or progressive cerebrovascular involvement, although further evidence is needed.
